# Microbial Resources and Enological Significance: Opportunities and Benefits

**DOI:** 10.3389/fmicb.2017.00995

**Published:** 2017-06-08

**Authors:** Leonardo Petruzzi, Vittorio Capozzi, Carmen Berbegal, Maria R. Corbo, Antonio Bevilacqua, Giuseppe Spano, Milena Sinigaglia

**Affiliations:** Department of the Science of Agriculture, Food and Environment, University of FoggiaFoggia, Italy

**Keywords:** wine, yeasts, non-*Saccharomyces*, lactic acid bacteria, microbial resources, starter cultures, alcoholic fermentation, malolactic fermentation

## Abstract

Among the innovative trends in the wine sector, the continuous exploration of enological properties associated with wine microbial resources represents a cornerstone driver of quality improvement. Since the advent of starter cultures technology, the attention has been focused on intraspecific biodiversity within the primary species responsible for alcoholic fermentation (*Saccharomyces cerevisiae*) and, subsequently, for the so-called ‘malolactic fermentation’ (*Oenococcus oeni*). However, in the last decade, a relevant number of studies proposed the enological exploitation of an increasing number of species (e.g., non-*Saccharomyces* yeasts) associated with spontaneous fermentation in wine. These new species/strains may provide technological solutions to specific problems and/or improve sensory characteristics, such as complexity, mouth-feel and flavors. This review offers an overview of the available information on the enological/protechnological significance of microbial resources associated with winemaking, summarizing the opportunities and the benefits associated with the enological exploitation of this microbial potential. We discuss proposed solutions to improve quality and safety of wines (e.g., alternative starter cultures, multistrains starter cultures) and future perspectives.

## Introduction

Wine has been consumed by humans for thousands of years and produced by crushing grapes and allowing them to ferment using the organisms present on the grapes and in the surrounding environment (Whitener et al., 2016). The microbiology of wines involves two main phases, alcoholic fermentation (AF) and malolactic fermentations (MLF) that rely on a heterogeneous microbiota composed by different indigenous microorganisms (e.g., yeast, bacteria and filamentous fungi). Considerable possible cellular/biochemical interactions can take place among these microbial resources in wine (Liu J. et al., 2017). Although of the entire wine microflora contribute to the wine chemistry, yeasts detain a predominant role (Capozzi et al., 2015). Yeast species are usually classified in two groups: *Saccharomyces* and non-*Saccharomyces* (Taillandier et al., 2014). *Saccharomyces* species play the key role, with *Saccharomyces cerevisiae* as the dominant species (Masneuf-Pomarède et al., 2010). On the other side, non-*Saccharomyces* yeast include different genera such as *Hanseniaspora*, *Issatchenkia*, *Pichia* and, *Schizosaccharomyces*, *Brettanomyces*, *Zygosaccharomyces*, *Kluyveromyces*, *Candida*, *Torulaspora* (Taillandier et al., 2014). These yeasts can experience anaerobic or aerobic growth and may persist during the fermentation, competing with *Saccharomyces* for nutrients, producing secondary compounds or modifying the metabolism of *S. cerevisiae* (Ciani et al., 2016). Several genera of lactic acid bacteria (LAB) have been reported in association to the wine prokaryotic consortia: *Enterococcus*, *Lactobacillus*, *Leuconostoc*, *Oenococcus*, *Pediococcus*, and *Weissella* (Capozzi et al., 2011; Berbegal et al., 2016; Salvetti et al., 2016; Cappello et al., 2017). In particular, *Oenococcus* *oeni* is the main species associated with MLF because of its tolerance to the harsh wine conditions (high ethanol concentration, low pH and nutrient content) (Cappello et al., 2017), even if increasing studies confirmed the relevance of *Lactobacillus plantarum* strains associated with specific physico-chemical conditions (du Toit et al., 2011).

Inoculation of selected starter cultures in wine must is an established enological practice in order to mitigate product losses or the production of off-flavors (García-Ríos et al., 2014; Whitener et al., 2015). Currently, a debate is still open about the use of commercial starters able to mimic some advantageous enological traits, which are present when the spontaneous fermentation is ruled by indigenous populations (Capozzi et al., 2015). For example, wines produced using single inocula are thought to lack the sensory complexity and rounded palate structure (Bellon et al., 2013). In this respect, mixed fermentation/multi-strains starter cultures including non-*Saccharomyces* yeasts/malolactic bacteria are generally regarded as having improved characteristics, such as complexity, mouth-feel and flavors (Tronchoni et al., 2017).

This review offers an overview of the available information on the enological/protechnological significance of microbial resources associated with winemaking, with a special focus on non-*Saccharomyces* yeast genera/species. A summary of the current state of knowledge about the microbial strategies to solve some technological and safety problems in winemaking is given in **Table 1**.

**Table 1:** Microbial strategies to solve some technological and safety problems in winemaking.

Specific application	Microbial resource(s)	Reference
Reducing volatile acidity	*Torulaspora delbrueckii*	Renault et al., 2009
	*Torulaspora delbrueckii* + *Saccharomyces cerevisiae*	Bely et al., 2008
	*Candida zemplinina* + *Saccharomyces cerevisiae*	Rantsiou et al., 2012
	*Candida stellata* + *Saccharomyces cerevisiae*	Ferraro et al., 2000
	*Hanseniaspora uvarum* + *Saccharomyces cerevisiae*	Tristezza et al., 2016
Alcohol reduction	*Metschnikowia pulcherrima* + *Saccharomyces cerevisiae*	Contreras et al., 2014
	*Torulaspora delbrueckii* + *Saccharomyces cerevisiae*	Belda et al., 2015
	*Candida stellata* + *Saccharomyces cerevisiae*	Ferraro et al., 2000
	*Candida zemplinina* + *Saccharomyces cerevisiae*	Bely et al., 2013
	*Saccharomyces cerevisiae* (genetically engineered yeast)	Varela et al., 2012
Modulation of acidity	*Schizosaccharomyces pombe* + *Lachancea thermotolerans*	Gobbi et al., 2013
	*Lachancea thermotolerans* + *Saccharomyces cerevisiae*	Benito A. et al., 2015
	*Schizosaccharomyces pombe*	Benito et al., 2016b
	*Oenococcus oeni*	Cappello et al., 2017
	*Lactobacillus plantarum*	Sun et al., 2016
Increased glycerol content	*Candida stellata* + *Saccharomyces cerevisiae*	Soden et al., 2000
	*Pichia fermentans* + *Saccharomyces cerevisiae*	Clemente-Jimenez et al., 2005
	*Candida zemplinina* + *Saccharomyces cerevisiae*	Englezos et al., 2016b
	*Lachancea thermotolerans* + *Saccharomyces cerevisiae*	Comitini et al., 2011
	*Saccharomyces cerevisiae* (genetically engineered yeast)	Dequin, 2011
Modulation of aroma profiles	*Torulaspora delbrueckii*	Renault et al., 2009
	*Saccharomyces bayanus*	Masneuf-Pomarède et al., 2010
	*Metschnikowia pulcherrima* + *Saccharomyces cerevisiae*	Sadoudi et al., 2012
	*Zygotorulaspora florentina* + *Saccharomyces cerevisiae*	Lencioni et al., 2016
	*Hanseniaspora vineae* + *Saccharomyces cerevisiae*	Viana et al., 2009
Enhancing varietal aromas	*Pichia kluyveri* and *Candida zemplinina*	Anfang et al., 2009
	*Saccharomyces cerevisiae*	Swiegers et al., 2009
	*Metschnikowia pulcherrima, Torulaspora delbrueckii*, and *Lachancea thermotolerans*	Zott et al., 2011
Mannoprotein release	*Saccharomyces cerevisiae* × *Saccharomyces kudriavzevii* (hybrids)	Pérez-Través et al., 2016
	*Torulaspora delbrueckii*	Belda et al., 2015
	*Saccharomyces cerevisiae* (genetically engineered yeast)	Gonzalez-Ramos et al., 2008
Control of spoilage microflora	*Torulaspora delbrueckii*	Comitini et al., 2017
	*Candida pyralidae*	Mehlomakulu et al., 2014
	*Lactobacillus plantarum*	Sun et al., 2016
Low sulphite formation	*Saccharomyces cerevisiae*	Balboa-Lagunero et al., 2013
Reduction of copper content	*Saccharomyces cerevisiae*	Sun et al., 2015
Reduction of ochratoxin A	*Saccharomyces cerevisiae*	Petruzzi et al., 2017
Reduced production of ethyl carbamate	*Schizosaccharomyces pombe*	Benito et al., 2016b
	*Saccharomyces cerevisiae* (recombinant strain)	Guo et al., 2016
Low biogenic amine formation	*Hanseniaspora vineae* + *Saccharomyces cerevisiae*	Medina et al., 2013
	*Schizosaccharomyces pombe*	Benito et al., 2016b


## *Saccharomyces cerevisiae* Model for Winemaking Workhorse

For the last two decades, the natural variation of the yeast *S. cerevisiae* has been massively exploited with the aim of understanding ecological and evolutionary processes (Cubillos, 2016). It has been used as a model to study aging, regulation of gene expression, signal transduction, cell cycle, metabolism, apoptosis, neurodegenerative disorders, and many other biological processes (Karathia et al., 2011). Genome sequence information is now available for >80 strains of *S. cerevisiae* in some form (complete, draft, or raw data) (Borneman and Pretorius, 2015). In addition, it is a key player in many industrial fermentation of food matrices such as wine, bread and sake (Cubillos, 2016). The first reported use of a selected yeast starter for wine production dates from 1890, when Müller-Thurgau introduced this technology adapting the techniques developed by Christian Hansen for the Carlsberg Brewery (Padilla et al., 2016). Since then, the role of *S. cerevisiae* as starter culture in the wine industry has received the most attention. This yeast is not only responsible for the metabolism of grape sugar to alcohol and CO_2_ but has other important side-roles, including the conversion of grape aroma precursors to sensory active molecules (Jolly et al., 2014; Belda et al., 2017). The metabolism of fermenting *S. cerevisiae* can be divided into primary and secondary. Primary metabolism is essential for growth, cell division and survival, producing metabolites such as ethanol, glycerol, acetaldehyde, and acetic acid. Secondary metabolites include the fusel alcohols, esters, carbonyls, sulfur compounds, thiols and terpenoids (Romano et al., 2015; Hirst and Richter, 2016). The dominance of *S. cerevisiae* in the fermentation is expected and desired (Jolly et al., 2014; Capece et al., 2016). One of the main features that allow *S. cerevisiae* to overcome is its remarkable sugar consumption rate and ethanol production coupled with a high alcohol tolerance. Through this quick proliferation in grape must *S. cerevisiae* efficiently depletes nitrogen sources and other nutrients required for yeast biomass production from the medium (Tronchoni et al., 2017). In addition, several studies raised evidence that microbial interactions play an important role in the early death of non-*Saccharomyces* yeasts (Albergaria and Arneborg, 2016). Therefore, *S. cerevisiae* has been the primary choice for producing wine starters (Albergaria and Arneborg, 2016). In modern winemaking, fermentations are driven largely by single-strain inoculations; pure cultures of selected strains of *S. cerevisiae* are added to grape must as soon as possible after crushing. This practice ensures the control of vinification, leads to outcomes that are more predictable and decreases the risk of spoilage by other microorganisms (Chambers and Pretorius, 2010). Several studies addressed the genetics underlying these unique properties and tried to unravel the evolutionary path *Saccharomyces* strains have undergone to become the specialized fermentation organisms they are today. It was shown that duplication of several key genes, such as those encoding alcohol dehydrogenase, hexose transporters, and enzymes linked to glycolysis, as well as global rewiring of the transcriptional network after whole genome duplication, might contribute to the suitability of *S. cerevisiae* as a driver of industrial fermentations (Steensels and Verstrepen, 2014). There are many-probably hundreds of-different yeast strains available, and the winemaker’s choice could have a significant effect on the quality of the wine (Chambers and Pretorius, 2010). **Table 2** shows the main enological properties of some exemplificative commercially available *S. cerevisiae* wine yeasts. However, while this practice may reduce sources of microbial spoilage, some winemakers feel that the exclusive use of *S. cerevisiae* has resulted in a lack of organoleptic complexity when compared with successful spontaneous fermentations (Whitener et al., 2015), thus contributing to an increased interest on the role of non-*Saccharomyces* yeasts in winemaking (Whitener et al., 2016).

**Table 2:** Main enological properties of some commercially available *S. cerevisiae* wine yeasts.

Commercial name	Feature(s) of interest in winemaking	Providing company
Merit^TM^	Is able to achieve alcoholic fermentation in high-alcohol wines (up to 16% vol.) or during the second alcoholic fermentation in sparkling wines.	Chr. Hansen (Hørsholm, Denmark)
Actiflore^®^ BO213	Extreme resistance to alcohol (18% vol.), with neutral characters and low SO_2_ production. Recommended for fermentation restart. Adapted to low temperature fermentations.	Laffort (Bordeaux, France)
WE372	Fermentation in cold temperature.	Anchor Yeast (Eppindust, South Africa)
Fermivin^®^ PDM	Sparkling wines (either for first or second fermentation).	DSM (Heanor, United Kingdom)
Levuline^®^ BRG^®^ Yseo^®^	Overproduction of mannoproteins.	Oenofrance (Reims, France)
Vitilevure^®^ MT^®^ YSEO^®^	Preservation of color of red wine.	Martin Vialatte (Reims, France)
Lalvin C^®^ Cross Evolution	Enhances the varietal character.	Lallemand (Montréal, QC, Canada)
Premier Cuvée	Tolerance to ethanol and free sulfur dioxide, and fermentation to dryness.	Red Star (Milwaukee, WI, United States)
AW4	Powerfully fragrant, full spice aromatic wines; is a perfect match for Gewurztraminer and recommended for Sauvignon and Semillon.	Vintner’s Harvest (Saskatoon, Canada)
Oenoferm^®^ F3 Rouge	Color preservation. This yeast is very suitable for red wines with pronounced fruit character.	Erbslöh (Geisenheim, Germany)
SafOEno^TM^ STG S101	It develops fruit (especially fermentative esters) and flower aromas; it is recommended for primeurs processed from carbonic maceration or thermovinification, as well as rosés. Wines have a fresh and light finish.	Fermentis (Marcq en Baroeul, France)
GV2	For full bodied red and white wines. Quick start, rapid ferment, low foam.	Muntons (Suffolk, United Kingdom)
WLP730	Slight ester production, low sulfur dioxide production. Enhances varietal character.	White Labs (San Diego, CA, United States)
4946 Bold Red/High Alcohol	Dominating, strong fermentation characteristics. Ideal for Zinfandel, Pinot Noir, Syrah, or any high sugar must. Good choice for restarting stuck fermentations.	Wyeast (Hood River, United States)
SIHA^®^ Aktivhefe 3	Quickly suppresses wild yeasts and bacteria, prevents unwanted fermentation side products. Produces clear wines with a prominent character (clear bouquet according to the variety and vineyard location).	Begerow (Langenlonsheim, Deutschland)
Ferm D20	Is recommended for the production of high-end red wines intended to be aged. It tolerates high fermentation temperatures, promotes extraction of phenolic compounds, and reduces the perception of green notes while enhancing aromatic intensity and complexity.	Enartis (Windsor, CA, United States)
Blastosel FR95	The aromatic profile is particularly rich and complex, with strong fruity notes to the fore completed by significant notes of rose.	Perdomini (Verona, Italy)


## Non-*Saccharomyces* Yeasts

The world wine market is experiencing increasing interest in new yeast strains that can produce unique wines with novel properties (Mylona et al., 2016). Non-*Saccharomyces* yeasts have garnered interest in winemaking due to their beneficial effects and because consumers are demanding new wine styles (Lleixà et al., 2016). Recently, some commercial yeast manufacturers have already included non-*Saccharomyces* yeasts as part of their product portfolio (Albergaria and Arneborg, 2016). The main enological properties of some commercially available non-*Saccharomyces* wine yeasts are outlined in **Table 3**. The main benefit of using complementary yeast starters in winemaking is the improved aromatic complexity (Comitini et al., 2011; Tronchoni et al., 2017). Non-*Saccharomyces* yeasts possess enzymatic activities, which can catalyze the release of volatile aroma compounds from non-volatile bound precursors. These yeasts can also affect aroma production directly by their own metabolic activity (production of alcohols and esters) or by the release of extracellular enzymes which transform *S. cerevisiae*-derived metabolites. Strains of non-*Saccharomyces* yeasts have also shown potential for producing aroma compounds not associated with fermentation by many strains of *S. cerevisiae*, such as various monoterpenes and other terpenoid compounds (Rossouw and Bauer, 2016). In addition, the use of non-*Saccharomyces* yeasts has been proposed to improve glycerol or mannoprotein content, volatile acidity, or color stability (Tronchoni et al., 2017) or to reduce the ethanol levels of wines (Rossouw and Bauer, 2016). On the other hand, there have been a large number of researches on the isolation and characterization of yeast strains degrading malic acid, as alternative to malolactic bacteria-fermentation starters (Kim et al., 2008). Positive features of non-*Saccharomyces* yeasts have been highlighted regarding to the production of metabolites beneficial for wine quality and stability. This is the case of yeasts producers of active extracellular molecules, able to counteract the development of wild spoilage microorganisms (Comitini et al., 2017).

**Table 3:** Main enological properties of some commercially available non-*Saccharomyces* wine yeasts.

Specie(s)	Commercial name	Feature(s) of interest in winemaking	Providing company
*Torulaspora delbrueckii*	Biodiva^TM^	Enhances aroma and mouthfeel complexity in white and red wines.	Lallemand
*Torulaspora delbrueckii*	Zymaflore^®^ Alpha^TD n. sacch^	Makes wines of high organoleptic complexity.	Laffort
*Torulaspora delbrueckii*	Prelude^TM^	Increases body, soft structure.	Chr. Hansen
*Torulaspora delbrueckii*	Oenoferm^®^ wild & pure [HR23]	Creamy texture with a pleasant and lasting mouthfeel.	Erbslòh
*Torulaspora delbrueckii*	WLP603	Provides aromatic complexity and a fresh fruit characteristics. Produces low volatile acids, volatile phenols, and ethyl acetate.	Vintner’s Harvest
*Lachancea thermotolerans*	Concerto^TM^	Produces lactic acid, giving roundness and balanced acidity to wines; suggested in warm regions.	Chr. Hansen
*Lachancea thermotolerans*, *Torulaspora delbrueckii* and *Saccharomyces cerevisiae*	Melody^TM^	Increases wine complexity. gives tropical fruitness and an overall aromatic intensity, combined with a round, balanced mouthfeel	Chr. Hansen
*Metschnikowia pulcherrima*	Flavia^®^	Enhances varietal aromas, terpenes and thiols aromas	Lallemand
*Metschnikowia fructicola*	Gaia^TM^	Selected for its ability to dominate the must during cold soak in order to offer a natural protection against spoilage microorganisms. The use of this yeast allows winemaker to reduce the SO_2_ at crushing.	Perdomini
*Pichia kluyveri*	WLP605	Produces rose petal and floral aromas, contributing to overall bouquet of wine.	Vintner’s Harvest
*Pichia kluyveri*	FrootZen^®^	Enhances varietal aromas, and thiols aromas.	Chr. Hansen
*Schizosaccharomyces pombe*	ProMalic^®^	Allows maloalcoholic deacidification.	Proenol


However, most non-*Saccharomyces* yeasts cannot ferment to dryness, thus *S. cerevisiae* should be also inoculated. Two modes of inoculation have been proposed and used (Whitener et al., 2016): the first is known as co-inoculation and some studies have showed that the inoculation of selected non-*Saccharomyces* yeasts at high cell concentration together with *S. cerevisiae* might produce wines with distinct characteristics while avoiding stuck fermentations. Others researchers have explored the use of non-*Saccharomyces* yeasts in sequential inoculations; non-*Saccharomyces* yeasts are first inoculated at high levels and allowed to ferment on their own for a given amount of time before *S. cerevisiae* is added to take over the fermentation. This practice gives the non-*Saccharomyces* yeast more time to express their unique metabolic footprint uninhibited by the stress of *Saccharomyces* competition (Whitener et al., 2015). Moreover, the effects of the non-*Saccharomyces* yeasts on fermentation and wine quality were strictly dependent on the *Saccharomyces*/non-*Saccharomyces* inoculum ratio (Comitini et al., 2011).

The principal outcomes of fermentations conducted with the aid of non-*Saccharomyces* yeast genera/species have been documented in the following subsections and summarized in Supplementary Table S1.

### Torulaspora delbrueckii

*Torulaspora delbrueckii* was one of the first commercial non-*Saccharomyces* yeast to be released (Jolly et al., 2014). This species has been previously suggested for the vinification of low sugar and acidity musts, and it has been used for the production of red and rosé wines in Italy and for Sauvignon Blanc in South Africa (González-Royo et al., 2015). *T. delbrueckii* is characterized by high purity fermentation, low production of glycerol, acetaldehyde, acetic acid, and ethyl acetate (Loira et al., 2015). When used in sequential or mixed fermentations with *S. cerevisiae*, it can contribute to correct certain defects such as the volatile acidity (Loira et al., 2015). In Semillon wine, a mixed *T. delbrueckii*/*S. cerevisiae* culture at a 20:1 ratio produced 53% less in volatile acidity and 60% less acetaldehyde than a pure culture of *S. cerevisiae* (Bely et al., 2008). Some authors showed that the strong β-glucosidase activity of this species enhanced wine aroma by modulating the levels of nor-isoprenoids, terpenols, and lactones by hydrolysing their respective precursors (Renault et al., 2015). Maturano et al. (2012) confirmed the high production of extracellular enzymes of enological relevance by this species. Amarone wines produced by co-inoculation/sequential inoculation with *T. delbrueckii* and *S. cerevisiae* were judged to have increased aroma intensity, including ‘ripe red fruit’ aroma, increased sweetness and astringency and decreased intensity for vegetal attributes (Azzolini et al., 2012). Gewurztraminer wines produced by sequential inoculation showed increased concentration of terpenes α-terpineol and linalool (Cus and Jenko, 2013). *T. delbrueckii*/*S. cerevisiae* multi starters have been proposed to modulate wine flavor in Sauvignon Blanc and Merlot (Renault et al., 2015), as well as Shiraz wines (Loira et al., 2015).

The growth of *T. delbrueckii* is often negatively affected by the presence of *S. cerevisiae*. Indirect interaction, i.e., interaction between strains via components of the medium have been demonstrated. Substrate competition or amensalism (production by *S. cerevisiae* of metabolites that inhibit *T. delbrueckii* growth) are examples of the indirect interactions already suggested (Taillandier et al., 2014).

Recently, new killer toxins from *T. delbrueckii* with potential biocontrol activity of *Brettanomyces bruxellensis*, *Pichiaguilliermondii*, *P. manshurica* and *P. membranifaciens* wine spoilage were identified and characterized (Comitini et al., 2017).

### Lachancea thermotolerans

*Lachancea thermotolerans* has been investigated for its ability to enhance wine acidity and improve the overall quality (Whitener et al., 2016). *L. thermotolerans* produces high concentration of L-lactic acid from glucose and fructose as well as low levels of volatile acidity and undesirable flavor compounds (Erten and Tanguler, 2010). This attribute could be of concern to address the problems of increased alcohol content and a reduction in the total acidity of wines associated with global climate change and variations in viticulture and wine-making practices (Balikci et al., 2016). *L. thermotolerans* was used as starter culture in Airén wine (increase of lactic acid of 3.18 g/L and pH reduction of 0.22) (Benito et al., 2016a), Emir wines (the use of *L. thermotolerans* in mixed and sequential cultures led to an increase in final total acidity of 5.40–6.28 g/L) (Balikci et al., 2016), Riesling wine (peach/apricot character) (Benito S. et al., 2015), Sangiovese and Cabernet-Sauvignon (spicy attributes) (Gobbi et al., 2013).

### Metschnikowia pulcherrima

*Metschnikowia pulcherrima* is commonly associated with grapes and wine (Whitener, 2016). It is a high producer of β-glucosidase and its presence in mixed cultures can decrease the volatile acidity and increase the production of medium-chain fatty acids, higher alcohol, esters, terpenols and glycerol (González-Royo et al., 2015). Wines of the grape varieties Chardonnay and Shiraz obtained by sequential fermentation with *Caesalpinia pulcherrima* and S showed ca. 1% v/v lower ethanol concentration (Varela et al., 2016). In addition, *M. pulcherrima* has been reported to increase: (1) the levels of methyl butyl-, methyl propyl-, and phenethyl esters production in Sauvignon Blanc (Whitener et al., 2016); (2) the ‘overall impression,’ ‘citrus/grape fruit’ and ‘pear’ attributes of Riesling (Benito S. et al., 2015); (3) foam persistence and ‘smoky’ and ‘flowery’ attributes of Macabeo wine (González-Royo et al., 2015). It has also been reported that *M. pulcherrima* might have an antagonistic effect toward several yeast. *cerevisiae*s including *S. cerevisiae* which leads to delays in fermentation. This phenomenon was the result of a pulcherrimin pigment (Jolly et al., 2014).

### *Candida* Species

The genus *Candida* is large and extremely diverse with over 50 different identified species several of which have been associated with winemaking (Whitener, 2016). Among the non-*Saccharomyces* wine yeasts involved in grape juice fermentation, *C. stellata* has been frequently isolated during the course of must fermentations in different countries (fermentation of botrytized wines and other wines produced by overripe grapes, in cooked musts, and in traditional balsamic vinegars) (Tofalo et al., 2012). Ciani and Maccarelli (1998) suggested that it might be used as a starter culture to increase glycerol levels in Trebbiano Toscano wine, where it 11.76 g/L of glycerol, which is higher than the sensory threshold level for glycerol sweetness, i.e., 5.2 g/L (Jolly et al., 2006). Similarly, wines of the grape variety Syrah obtained by mixed and sequential fermentation with *C. cantarellii* and *S. cerevisiae* showed 44.3 to 52.8% glycerol content higher than the control wines (Toro and Vazquez, 2002). *C. sake* increased concentrations of terpenes and higher alcohols in Pedro Gimenez wines (Maturano et al., 2015).

*C. zemplinina* (synonym *Starmerella bacillaris*; Duarte et al., 2012) is a non-*Saccharomyces* yeast, isolated for the first time in Napa Valley (California, United States) in 2002, under the name EJ1 (Mills et al., 2002). This yeast differs from the other common non-*Saccharomyces* yeasts, since it can survive and resist until the end of the AF due to its ability to tolerate high concentrations of ethanol (Englezos et al., 2016a).

*Candida zemplinina* showed some interesting characteristics, such as: (1) high glycerol production (Tofalo et al., 2012; Zara et al., 2014; Englezos et al., 2016b); (2) reduced ethanol yield (Bely et al., 2013; Englezos et al., 2016b); (3) increase of aroma complexity (Tofalo et al., 2016; Whitener et al., 2016); (4) ability to metabolize malic acid (Tofalo et al., 2012); (5) reduction of the acetic acid production in combination with *S. cerevisiae* (Rantsiou et al., 2012). These applications support the use of *C. zemplinina*, which could be a good choice to achieve various desired results, mainly due to its fructophilic character and the poor ethanol yield from sugar (Masneuf-Pomarede et al., 2015; Englezos et al., 2016a).

Recently, Mehlomakulu et al. (2014) found two novel killer toxins, CpKT1 and CpKT2, from the wine isolated strains of *C. pyralidae*, able to control the development of spoilage yeast *B. bruxellensis*. In addition, these killer toxins inhibited neither the *Saccharomyces cerevisiae* nor the LAB strains tested.

### *Pichia* Species

*Pichia fermentans* was investigated by Clemente-Jimenez et al. (2005) in lab-scale vinifications of Macabeo wine. Sequential fermentations with *P. fermentans* and *S. cerevisiae* produced wines with increased concentrations of acetaldehyde, ethyl acetate, 1-propanol, *n*-butanol, 1-hexanol, ethyl caprilate, 2,3-butanediol and glycerol.

Sequential inoculum of Riesling must with *P. kluyveri* and *S. cerevisiae* increased the ‘overall impression’ and ‘peach/apricot’ character (Benito S. et al., 2015). Co-fermentation of Sauvignon Blanc grape juice with *P. kluyveri* has been reported to lead to higher levels of 3-mercaptohexyl acetate (Anfang et al., 2009); however, it has also been reported to produce many off odor compounds (Whitener et al., 2016).

Malic acid is sometimes detrimental to the quality of wines when present at high concentrations in some varieties. Several grape varieties contain considerable amounts of malic acid and grapes grown in the cooler regions contain higher amounts of malic acid than those grown in the warmer regions. Excessive amounts of malic acid (15–16 mg/ml) were detected in grapes grown during exceptionally cold summer in cool regions (Kim et al., 2008). In this respect, co-inoculation of *P. kudriavzevii* enhance the catalysis of malic acid in grape juice fermentation (Kim et al., 2008).

It is reported that *P. membranifaciens* increased esters production in Muscat wine (Viana et al., 2008).

### Schizosaccharomyces pombe

*Schizosaccharomyces pombe* was initially considered as a spoilage yeast because of the production of undesirable metabolites with a negative sensory impact; on the other hand, it could be successfully used at an industrial level in cane sugar fermentation during rum making, palm wine production and cocoa fermentation (Mylona et al., 2016). However, *Schizosaccharomyces pombe* species is highly appreciated in colder regions because of its ability to completely transform the malic acid of the must into ethanol, thanks to its particular metabolism of maloalcoholic fermentation (Loira et al., 2015). In this respect, *S. pombe* perform effective malic acid deacidification and significantly reduces the levels of biogenic amines and ethyl carbamate precursors without the need for any secondary bacterial MLF (Benito et al., 2014, 2016b). Mylona et al. (2016) confirmed the ability of yeast to produce safe wines.

One new application exploits *Schizosaccharomyces* ability to increase the formation of vitisins and vinylphenolic pyranoanthocyanin (Loira et al., 2015). In addition, the rapid autolytic release of cell wall polysaccharides after death could reduce the time required to complete aging over lees (Benito et al., 2014).

### *Hanseniaspora* Species

The apiculate yeast *Hanseniaspora uvarum* is the non-*Saccharomyces* found at highest levels in grape must, in fact it is among the relevant contributors to wine quality (Jolly et al., 2014). In single fermentation, *H. uvarum* produced low volatile acidity and high levels of glycerol (Tofalo et al., 2016). *H. uvarum* has also been used in mixed fermentations with *S. cerevisiae* for wine production. Tristezza et al. (2016) assessed the oenological potential of *H. uvarum* in co-inoculation and in a sequential inoculation with *S. cerevisiae* for industrial wine production. The mixed starter was able to successfully dominate the different stages of the fermentation process and the *H. uvarum* strain ITEM8795 contributed to increase the wine organoleptic quality and reduce the volatile acidity. In Solaris wine, mixed fermentations produced higher levels of glycerol, heptyl acetate, and 2-phenylethyl acetate (Liu et al., 2016).

*Hanseniaspora vineae* has been demonstrated to increase fruity aromas and produce high amounts of acetate esters, such as 2-phenylethyl acetate and ethyl acetate, in wines produced by a sequential fermentation with *S. cerevisiae* (Lleixà et al., 2016). Co-fermentation of Bobal grape must with *H. vineae* and *S. cerevisiae* produced wines that not only showed an increased concentration of 2-phenylethyl acetate (approximately three–ninefold higher) but also exhibited higher ‘fruity’ sensory scores than wines produced with *S. cerevisiae* pure culture (Viana et al., 2009). Chardonnay wines produced with *H. vineae* and *S. cerevisiae* have shown increased aroma and flavor diversity and reduced biogenic amines content (Medina et al., 2013).

*Hanseniaspora guilliermondii* can contribute to the overall quality of wines. In single-culture this yeast produced high amounts of 2-phenylethyl acetate (Viana et al., 2008). Tinta Roriz grape must inoculated with *H. guilliermondii* led to the production of wine with higher concentrations of 1-propanol, 2-phenylethyl acetate and 3-(methylthio)propionic acid, and lower amounts of ethyl hexanoate, pentanoic acid, free fatty acids, 2-methyltetrahydrothiophen-3-one and acetic acid-3-(methylthio)propyl ester (Moreira et al., 2011).

### *Zygosaccharomyces* Species

The genus *Zygosaccharomyces* is known, together with *Brettanomyces* species (Capozzi et al., 2016a), for its ability to spoil wine, namely sweet and sparkling wines (Whitener et al., 2016). *Zygosaccharomyces bailii* and *Zygosaccharomyces rouxii* are often the source of spoilage in acidic and shelf-stable foods as well as sweet wines due to their ability to tolerate high acid, salt and sugar conditions (Whitener, 2016). However, selected strains of *Zygosaccharomyces* spp. might be useful (Garavaglia et al., 2015), because they can yield high levels of ethyl esters in Chardonnay wine (Garavaglia et al., 2015). Ethyl esters are highly interesting because they have a pleasant fruity and floral aromatic note, and are responsible for beer and wine aroma (Garavaglia et al., 2015).

*Zygosaccharomyces bailii* is fructophilic, and metabolizes fructose more easily than glucose (Garavaglia et al., 2015). This triat could be beneficial in grape musts from over-ripened grapes (Jolly et al., 2006). Zuehlke et al. (2015) used inoculation with *Z. bailii* to remove residual sugar from Cabernet Sauvignon and Syrah sluggish fermentations.

*Zygosaccharomyces kombuchaensis* is a newly discovered yeast. It can contribute to increase flavor intensity’ and several ‘fruity’ attributes in Ribolla Gialla (Dashko et al., 2015), as well as benzaldehyde in Sauvignon Blanc and Syrah (Whitener et al., 2015) wines.

### *Debaryomyces* Species

*Debaryomyces* may contribute to the maturation, aroma, and flavor of foods, such as cheese and meat products, but it also might spoil fermented food products (Wrent et al., 2014). In winemaking, two species are of interest: *Debaryomyces pseudopolymorphus* and *Debaryomyces vanriji*. Co-fermentation of Chardonnay grape juice with *D. pseudopolymorphus* and *S. cerevisiae* resulted in an increased concentration of the terpenols (citronellol, nerol, and geraniol) (Cordero Otero et al., 2003). Sequential fermentation of Muscat of Frontignan grape juice with *D. vanriji* and *S. cerevisiae* produced wines with increased concentration of geraniol (Garcia et al., 2002). Sequential fermentation of Pedro Gimenez must with *D. vanriji* and *S. cerevisiae* increased the concentrations of esters and fatty acids (Maturano et al., 2015).

### *Kazachstania* Species

The *Kazachstania* genus as a whole is fairly new; it was first mentioned in literature in 2003. *Kazachstania aerobia* was first identified in 2004 from corn silage while *Kazachstania gamospora* was discovered as a species in 2007. Being relatively new yeasts and closely related to *S. cerevisiae*, the genus is worthy of investigation (Whitener, 2016).

Dashko et al. (2015) used *K. gamospora* in Ribolla Gialla fermentation, resulting in increased acetate and ethyl ester amounts. In Sauvignon blanc and Syrah musts, this yeast caused a 200-fold increase of phenethyl propionate (Whitener et al., 2015). Sauvignon Blanc wines produced with *K. aerobia* and *S. cerevisiae* have shown increased ethyl acetate production (Whitener et al., 2016).

### Wickerhamomyces anomalus

*Wickerhamomyces anomalus* is a constituent of the normal grape flora in the early phases of fermentation. It could spoil wine by excessive production of acetic acid and ethyl acetate, but also contributes to wine aroma by the production of volatile compounds. This species has gained considerable biotechnological interest due to its tolerance toward environmental stress factors (e.g., low pH, high osmolarity), metabolic versatility and production of exoenzymes (Sabel et al., 2014). Mazuela wines produced by sequential inoculation of *W. anomalus* and *S. cerevisiae* showed increased concentration of acetate- and ethyl- esters, along with a significant and panel preference (Cañas et al., 2014).

### Williopsis saturnus

*Williopsis saturnus* has been reported to increase the levels of main terpenols (linalool, citronellol, and α-terpineol), produce some terpenoid esters (citronellyl and neryl acetate) and retain the concentration of *cis*-rose oxide in mango wine with ethanol levels of 2–4% (v/v) (Chen et al., 2015). This specie is not generally found from the natural environment of surfaces of grapes and winery equipments: however, it might potentially enhance the fruity flavor in wines obtained from neutral cultivar characteristics (Erten and Tanguler, 2010).

Emir wines produced by co-inoculation with *W. saturnus* and *S. cerevisiae* showed higher concentrations of acetic acid, ethyl acetate and isoamyl acetate (Erten and Tanguler, 2010).

### Zygotorulaspora florentina

*Zygotorulaspora florentina* was used in mixed fermentations at different inoculum ratio with *S. cerevisiae*, and caused an increase of the production of polysaccharides and a modulation of the final concentrations of the various volatile compounds (Domizio et al., 2011). Recently, Sangiovese wines produced by co-inoculation with *Zygotorulaspora florentina* and *S. cerevisiae* showed an enhancement of polysaccharides and 2-phenylethanol content, a reduction of volatile acidity, and high concentration of glycerol and esters (Lencioni et al., 2016).

## Bacteria

Malolactic fermentations is a secondary microbial-based biochemism that usually takes place in wine during or at the end of AF; MLF is mainly carried out by one or more species of LAB (Lerm et al., 2011; Cappello et al., 2017). During this phase, the biological conversion of interest in enology is the L-malic acid decarboxylation to produce L-lactic acid and carbon dioxide. Aside from impacts on acidity, LAB can also metabolize other precursors present in wine during fermentation and, therefore, affect the chemical composition of the wine resulting in an increased complexity of wine aroma and flavor (Sumby et al., 2014; Campbell-Sills et al., 2016).

Spontaneous MLF implies several risks, such as a considerable increase in volatile acidity, the consumption of residual sugars, and the formation of undesirable metabolites, such as biogenic amines, that can affect human health and lead to low quality wines (Spano et al., 2010). In recent years, wine industries have moved toward using pure starter cultures of selected LAB to promote a reliable and rapid malic acid bioconversion (Spano et al., 2010). **Table 4** shows the main enological properties of exemplificative commercially available wine malolactic bacteria.

**Table 4:** Main enological properties of some commercially available wine malolactic bacteria.

Specie(s)	Commercial name	Feature(s) of interest in winemaking	Providing company
*Oenococcus oeni*	Bactelia Crescendo	Can perform MLF under the most difficult winemaking conditions.	Oenofrance
*Oenococcus oeni*	Viniflora^®^ Oenos^TM^	Produces a medium amount of diacetyl.	Chr. Hansen
*Oenococcus oeni*	450 PreAc^®^	Specifically selected for high alcohol wines.	Laffort
*Oenococcus oeni*	Vitilactic Primeur^TM^	Selected for easily and effectively carrying out MLF on red wines.	Martin Vialatte
*Oenococcus oeni*	Lalvin VP41^TM^	Can perform under the most difficult winemaking conditions. It is recognized for its sensory contribution to red berry fruit aroma, its late and slow degradation of citric acid and very low production of diacetyl.	Lallemand
*Oenococcus oeni*	Ey2D	Suggested for white wines, and selected for its tolerance to low cellar temperatures.	Wyeast
*Oenococcus oeni*	Bi-Start^®^ Vitale SK11	Enhances the typical red wine character with very pronounced jam, cherry or ripe paprika flavors.	Erbslöh
*Oenococcus oeni*	WLP675	Produces moderate levels of diacetyl. Has a high tolerance to low pH (3.0), low temperature environments (down to 55°F or 12°C), and high alcohol percentages (up to 15% alcohol by volume).	Vintner’s Harvest
*Oenococcus oeni*	Sihalact^TM^ Oeno	Produces low concentration of diacetyl. High alcohol tolerance up to 15 vol.%.	Begerow
*Oenococcus oeni*	ML One	It produces clean and fruit forward aromas and helps reducing the impact of herbaceous notes that are sometimes present in red wines.	Enartis
*Lactobacillus plantarum*	Viniflora^®^ Nova^TM^	Is ideal for low-malic-acid must. Increases fruity aroma and flavor, especially red and blackberry attributes.	Chr. Hansen
*Lactobacillus plantarum*	V22^TM^	Recommended for high pH must. The strain has proved to result in a high expression of dark and red fruits in red wine. It can also degrade ochratoxin A in wine.	Lallemand
*Lactobacillus plantarum*, *Oenococcus oeni*	Anchor NT 202 Co-Inoculant	Enhances fruitiness of wines by producing esters that reduce the vegetative characters.	Anchor Yeast


Actually, the production of efficient malolactic starter cultures has become one of the main challenges for oenological research (Lerm et al., 2011; Berbegal et al., 2016; Cappello et al., 2017). There are various important criteria to address when selecting LAB for possible use in a starter culture, like the ability to tolerate low pH, high ethanol and SO_2_ concentrations, good growth characteristics under winemaking conditions, compatibility with the selected yeast strain, the inability to produce biogenic amines and the lack of off-flavor or off-odor production (Capozzi et al., 2010; Lerm et al., 2011). A minor but also important aspect to be considered is the susceptibility of LAB to polyphenols. *O. oeni* is the major LAB used in commercial starter cultures for MLF (Lerm et al., 2011). *O. oeni* has been the only species within the *Oenococcus* genus until the mid-2000s when *O. kitaharae* was identified in composting distilled shochu residue. Over centuries of selective pressure, *O. oeni* has honed and perfected various adaptive strategies that enable it to outcompete with other potential MLF bacteria, during the later stages of vinification and thus to dominate in wine (Campbell-Sills et al., 2015). Recently a third *Oenococcus* species has been identified, *O. alcoholitolerans* isolated from Brazilian cachaça (Cappello et al., 2017).

In the selection of MLF starters, a challenge is the time of inoculation. Starter cultures can be co-inoculated with yeast (at the beginning or toward the end of AF), or sequentially (after AF) (Bartowsky et al., 2015). Generally, it has been demonstrated that bacteria inoculated in must performed better than those inoculated after AF, especially when cell growth conditions are not favorable (Azzolini et al., 2010). Some *Lactobacillus* species have also showed the ability to survive the harsh wine conditions; the species *Lactobacillus plantarum* has shown the most potential as a starter culture (Lerm et al., 2011). This versatile bacterium tolerates ethanol up to 14% v/v and has similar SO_2_ tolerance of *O. oeni* (Cappello et al., 2017). The introduction of some *L. plantarum* strains to the fermenting musts could significantly modify the wine aroma profile due to a different enzymatic profile. In addition, *L. plantarum* could synthesize antimicrobial peptides which might help preventing the production of undesired compounds, or inhibiting the indigenous LAB microflora (Sun et al., 2016). Due to these characteristics, selected strains of *L. plantarum* are currently being commercialized to induce MLF in wine (**Table 4**).

## Concluding Remarks

Wine fermentation is generally performed through inoculated or spontaneous fermentation (Martiniuk et al., 2016). One of the main trends in the industry of starter cultures for enology relies on the survey of the microbial resources associated with spontaneous fermentation in order to design products able to maximize wine quality (Bokulich et al., 2016; Corbo et al., 2017; Romano and Capece, 2017; Russo et al., 2017). In this light, our contribute proposes an overview of the opportunities and benefits associated with the exploitation of this microbial potential in winemaking. Considering future perspectives, the increasing number of species/strains used, often associated to new isolations from spontaneous fermentations (e.g., Garofalo et al., 2015; Garofalo et al., 2016), introduces a relevant change in terms of interspecific interactions (Ciani et al., 2016; Liu Y. et al., 2017; Tronchoni et al., 2017). A field of particular interest if we consider that different grape juices and batch volumes could influence the growth and final biomass of yeasts in mixed fermentations (Gobbi et al., 2013) and that most studies have been performed at laboratory scale without an effective validation at industrial or semi-industrial scale, questioning their applicability at cellar (Belda et al., 2016). Moreover, it is relevant to underline how this protechnological potential is often a reservoir of interesting biotechnological applications in the food sector (Capozzi et al., 2011, 2016b; Russo et al., 2016; Petruzzi et al., 2017).

## Author Contributions

LP, VC, CB, GS, MC, MS, and AB performed an accurate research in the literature and planned paper. LP wrote the paper. LP, VC, CB, GS, MC, MS, and AB revised the manuscript.

## Conflict of Interest Statement

The authors declare that the research was conducted in the absence of any commercial or financial relationships that could be construed as a potential conflict of interest.
