# Supplementary material for: Microbial Resources and Enological Significance: Opportunities and Benefits
Source: Front Microbiol. 2017 Jun 8;8:995. doi: 10.3389/fmicb.2017.00995 (PMC5462979; doi:10.3389/fmicb.2017.00995)
Supplement: Supplementary file 1 [file Table_1.docx]

Supplementary Material

Microbial resources and enological significance: opportunities and benefits

Leonardo Petruzzi^1^, Vittorio Capozzi^1^, Carmen Berbegal^1^ , Maria Rosaria Corbo^1^, Antonio Bevilacqua^1^, Giuseppe Spano^1*^, Milena Sinigaglia^1^

^1^Department of the Science of Agriculture, Food and Environment, University of Foggia Foggia, Italy

*** Correspondence:**Corresponding Author
[giuseppe.spano@unifg.it](mailto:giuseppe.spano@unifg.it)

# Supplementary Figures and Tables

For more information on Supplementary Material and for details on the different file types accepted, please see [here](http://home.frontiersin.org/about/author-guidelines#SupplementaryMaterial). **Table S1: Selected references on single and mixed starter cultures composed by *Saccharomyces* sp. and non-*Saccharomyces* yeasts**

| **Yeast specie** | **Inoculation regime** | **Scale of fermentation** | **Must** | **Feature(s) of interest in winemaking** | **Reference** |
| --- | --- | --- | --- | --- | --- |
| *Torulaspora delbrueckii* | Co-inoculation/ sequential | Lab-scale | Shiraz | An increase to the fruity character. | Loira et al. (2015) |
| *Torulaspora delbrueckii* | Single | Lab-scale | Malvar | High acetate esters and medium-chain fatty acids content. | Cordero-Bueso et al. (2013) |
| *Torulaspora delbrueckii* | Sequential | Pilot-scale | Macabeo | Increased foamability and foam persistence. | González‑Royo et al. (2015) |
| *Torulaspora delbrueckii* | Sequential | Medium-scale | Gewurztraminer | Increased concentration of terpenes α-terpineol and linalool. | Cus and Jenko (2013) |
| *Torulaspora delbrueckii* | Co-inoculation/ sequential | Lab-scale | Sauvignon blanc | Increased concentration of thiols, 3-sulfanylhexan-1-ol and 3-sulfanylhexyl acetate . | Renault et al. (2016) |
| *Torulaspora delbrueckii* | Co-inoculation/ sequential | Lab-scale/Pilot-scale | Sauvignon blanc/Merlot | Increase of esters production. Increased ‘complexity’ and ‘fruity’ attributes. | Renault et al. (2015) |
| *Torulaspora delbrueckii* | Co-inoculation/ sequential | Lab-scale | Semillon | A mixed *T. delbrueckii*/*S. cerevisiae* culture at a 20:1 ratio produced 53% less in volatile acidity and 60% less acetaldehyde than a pure culture of *S. cerevisiae*. | Bely et al. (2008) |
| *Torulaspora delbrueckii* | Co-inoculation/ sequential | Pilot-scale | Amarone | Increased aroma and complexity. | Azzolini et al. (2012) |
| *Torulaspora delbrueckii* | Co-inoculation/ sequential | Medium-scale/Pilot-scale | Tempranillo | The ethanol concentration was reduced; some malic acid was consumed; more pyruvic acid was released, and fewer amounts of higher alcohols were produced. | Belda et al. (2015) |
| *Torulaspora delbrueckii* | Single/ Co-inoculation | Lab-scale | Pedro Gimenez | High production of extracellular enzymes of enological relevance. | Maturano et al. (2012) |
| *Lachancea thermotolerans* | Co-inoculation/ Sequential | Lab-scale | Airén | The acidification process caused a lactic acid increment of 3.18 g/L and a reduction of 0.22 in pH compared to the control fermentation, performed by *S. cerevisiae*. | Benito et al. (2016a) |
| *Lachancea thermotolerans* | Co-inoculation | Lab-scale | N.I. | Glycerol overproduction. Acetate ester production. Reducing the pH, and enhancing the total acidity. | Comitini et al. (2011) |
| *Lachancea thermotolerans* | Sequential | Medium-scale | Riesling | Increased ‘overall impression’ and ‘peach/apricot’ characters. | Benito et al. (2015) |
| *Lachancea thermotolerans* | Single/Co-inoculation/ Sequential | Lab-scale/Industry-scale | Sangiovese, Cabernet-Sauvignon | Enhanced acidity and increased ‘spicy’ attributes. | Gobbi et al. (2013) |
| *Lachancea thermotolerans* | Single/Co-inoculation/ Sequential | Lab-scale | Emir | The use of *L. thermotolerans* in mixed and sequential cultures led to an increase in final total acidity content in the wines, varying in the range 5.40–6.28 g/l (as tartaric acid), compared to pure culture *S. cerevisiae*, which gave the lowest level of total acidity (5 g/l). High sensory scores. | Balikci et al. (2016) |
| *Metschnikowia pulcherrima* | Sequential | Lab-scale | Chardonnay and Shiraz | Wines showed approximately 1% v/v lower ethanol concentration than control (*S. cerevisiae* alone). | Varela et al. (2016) |
| *Metschnikowia pulcherrima* | Co-inoculation | Lab-scale | Sauvignon  Blanc | Aromas and esters production. | Zott et al. (2011) |
| *Metschnikowia pulcherrima* | Sequential | Lab-scale | Sauvignon Blanc | Higher level of aromatic compounds than the sum of the aromatic compounds present in each mono-culture, independent of biomass. | Sadoudi et al. (2012) |
| *Metschnikowia pulcherrima* | Sequential | Medium-scale | Sauvignon Blanc | High levels of methyl butyl-, methyl propyl-, and phenethyl esters production. | Beckner Whitener et al. (2016) |
| *Metschnikowia pulcherrima* | Sequential | Lab-scale | Muscat d’Alexandrie | α-Terpineol increase. | Rodríguez et al. (2010) |
| *Metschnikowia pulcherrima* | Sequential | Lab-scale/ Pilot-scale | Tempranillo | Improved clariﬁcation and phenolic extraction. | Belda et al. (2016) |
| *Metschnikowia pulcherrima* | Sequential | Medium-scale | Riesling | Increased ‘overall impression’, ‘citrus/grape fruit’ and ‘pear’ attributes. | Benito et al. (2015) |
| *Metschnikowia pulcherrima* | Sequential | Pilot-scale | Macabeo | Increased foam persistence and ‘smoky’ and ‘flowery’ attributes. | González‑Royo et al. (2015) |
| *Candida cantarellii* | Single/Sequential/Co-inoculation | Lab-scale | Syrah | In mixed and sequential cultures, the glycerol content of the ﬁnal products was 44.3 to 52.8% higher than the one obtained with pure *S. cerevisiae* fermentation. | Toro and Vazquez (2002) |
| *Candida sake* | Sequential | Medium-scale | Pedro Gimenez | Increased concentrations of terpenes and higher alcohols. | Maturano et al. (2015) |
| *Candida stellata* | Single | Lab-scale | Trebbiano Toscano | High glycerol production (average = 11:76 g/l). | Ciani and Maccarelli (1998) |
| *Candida stellata* | Sequential | Pilot-scale | Trebbiano Toscano | High glycerol production. Reduction in acetic acid and ethanol concentration. | Ferraro  et al. (2000) |
| *Candida stellata* | Single | Lab-scale | Furmint | Strong fructophilic character. High glycerol production. | Magyar and Tóth (2011) |
| *Candida stellata* | Sequential/Co-inoculation | Lab-scale | Pinot grigio | Increased glycerol content. A decrease in acetic acid and higher alcohols and an increase in succinic acid. | Ciani  and Ferraro (1998) |
| *Candida zemplinina* | Single | Lab-scale | Montepulciano  d’Abruzzo | Fructophily. Low ethanol and acetic acid production, high glycerol production, capacity to metabolize malic acid and slower fermentation kinetics when compared to *S. cerevisiae*. | Tofalo et al. (2012) |
| *Candida zemplinina* | Co-inoculation/Sequential | Lab-scale | Merlot | Reduced ethanol production. | Bely et al. (2013) |
| *Candida zemplinina* | Sequential | Medium scale | Sauvignon blanc | High number of terpenes in high concentration but also produced a large amount of acetic acid. | Beckner Whitener et al. (2016) |
| *Candida zemplinina* | Co-inoculation | Lab-scale/Pilot-scale | Bovale | Increased glycerol content, ‘jam’, ‘softness’, ‘structure’ and ‘persistence’ attributes, and decreased ‘bitter’ characters | Zara et al. (2014) |
| *Candida zemplinina* | Co-inoculation | Pilot-scale | Montepulciano d'Abruzzo | Increased ‘persistence’ and decreased ‘reduced’ characters | Tofalo et al. (2016) |
| *Candida zemplinina* | Co-inoculation/Sequential | Lab-scale and pilot-scale | Montepulciano | Laboratory scale fermentations demonstrated a decrease up to 0.7 % (v/v) of ethanol and an increase of about 4.2 g/L of glycerol when *S. cerevisiae* was inoculated with a delay of 48 h with respect to the inoculation of *S. bacillaris*. Pilot-scale fermentations confirmed the laboratory results. | Englezos et al. (2016b) |
| *Candida zemplinina* | Co-inoculation | Lab-scale | Barbera | Increased glycerol concentration, greater volatile complexity. | Englezos et al. (2016c) |
| *Pichia fermentans* | Single/sequential | Lab-scale | Macabeo | Sequential mixtures increase acetaldehyde, ethyl acetate, 1-propanol, n-butanol, 1-hexanol, ethyl caprilate, 2,3-butanediol and glycerol. | Clemente-Jimenez et al. (2005) |
| *Pichia kluyveri* | Co-inoculation | Lab-scale | Sauvignon Blanc | 3-mercaptohexyl acetate increase. | Anfang et al. (2009) |
| *Pichia kluyveri* | Sequential | Medium-scale | Sauvignon Blanc | Production of many off odor compounds. | Beckner Whitener et al. (2016) |
| *Pichia kluyveri* | Sequential | Medium-scale | Riesling | Increased ‘overall impression’ and ‘peach/apricot’ characters. | Benito et al. (2015) |
| *Pichia kudriavzevii* | Co-inoculation | Medium-scale | Campbell’s Early | Enhance the catalysis of malic acid. | Kim et al. (2008) |
| *Pichia membranifaciens* | Single | Lab-scale | Muscat | Esters production. | Viana et al. (2008) |
| *Schizosaccharomyces pombe* | Co-inoculation/ sequential | Lab-scale | Shiraz | Increased formation of vitisins and vinylphenolic pyranoanthocyanin. | Loira et al. (2015) |
| *Schizosaccharomyces pombe* | Single/Co-inoculation/ sequential | Lab-scale | Garnacha | Wine deacidiﬁcation. | Benito et al. (2014) |
| *Schizosaccharomyces pombe* | Single/ Co-inoculation | Lab-scale | Tempranillo | Lower acetic acid production and histamine content, a higher anthocyanin content and a higher color intensity. | Mylona et al. (2016) |
| *Schizosaccharomyces* *pombe* | Single | Lab-scale | N.I. | Reducing levels of biogenic amines and ethyl carbamate precursors. | Benito et al. (2016b) |
| *Hanseniaspora guilliermondii* | Single | Lab-scale | Muscat | Strong production of 2-phenylethyl acetate. | Viana et al. (2008) |
| *Hanseniaspora guilliermondii* | Single | Pilot scale | Tinta Roriz | The grape must inoculated with *H. guilliermondii* led to the production of wine with higher concentrations of 1 propanol, 2-phenylethyl acetate and 3-(methylthio)propionic acid, and lower amounts of ethyl hexanoate, pentanoic acid, free fatty acids, 2-methyltetrahydrothiophen-3-one and acetic acid-3-(methylthio)propyl ester, than wine resulting from the spontaneous fermentation. | Moreira et al. (2011) |
| *Hanseniaspora uvarum* | Single | Pilot scale | Montepulciano d'Abruzzo | Low volatile acidity. High levels of glycerol. | Tofalo et al. (2016) |
| *Hanseniaspora uvarum* | Sequential/Co-inoculation | Lab-scale, Pilot- and Industrial-scale | Negroamaro | The mixed starter contributed to increasing the wine organoleptic quality and to simultaneously reduce the volatile acidity. | Tristezza et al. (2016) |
| *Hanseniaspora uvarum* | Sequential | Lab-scale | Solaris | Mixed fermentations produced a larger amount of glycerol as well as heptyl acetate and 2-phenylethyl acetate. | Liu et al. (2016) |
| *Hanseniaspora vineae* | Co-inoculation | Lab-scale | Bobal | The concentration of 2-phenylethyl acetate was approximately 3- to 9-fold greater than that produced by *S. cerevisiae* pure culture. Moreover, sensory evaluation revealed a stronger fruity character in wines fermented with mixed cultures than in control wines. | Viana et al. (2009) |
| *Hanseniaspora vineae* | Sequential/Co-inoculation | Lab-scale | Tempranillo | Strong acetate ester production. | Viana et al. (2011) |
| *Hanseniaspora vineae* | Sequential | Pilot-scale | Chardonnay | Increased aroma and ﬂavour diversity compared with wines resulting from inoculation with *S. cerevisiae* alone. Reducing biogenic amines content. | Medina et al. (2013) |
| *Hanseniaspora vineae* | Co-inoculation | Pilot-scale | Macabeo | Increased concentration of phenyl-ethyl acetate, ethyl lactate and α-terpineol. | Lleixà et al. (2016) |
| *Hanseniaspora vineae* | Single/ Co-inoculation | Lab-scale | Pedro Gimenez | High production of extracellular enzymes of enological relevance. | Maturano et al. (2012) |
| *Zygosaccharomyces bailii* | Co-inoculation | Lab-scale | Chardonnay | Increased concentration of ethyl esters. | Garavaglia et al. (2015) |
| *Zygosaccharomyces bailii* | Single/ Co-inoculation | Lab-scale | Cabernet Sauvignon, Syrah | Yeast metabolized residual fructose in wines of varying alcohol content. | Zuehlke et al. (2015) |
| *Zygosaccharomyces kombuchaensis* | Sequential | Lab-scale/Pilot-scale | Ribolla Gialla | Increased ‘flavour intensity’ and several ‘fruity’ attributes. | Dashko et al. (2015) |
| *Zygosaccharomyces kombuchaensis* | Single | Lab-scale | Sauvignon blanc  and Syrah | Of the compounds that did show signiﬁcant fold increases the largest were benzaldehyde in both musts at 13 and 15 times more in the Sauvignon blanc and Syrah musts respectively. | Beckner Whitener et al. (2015) |
| *Debaryomyces vanrijiae* | Sequential | Medium-scale | Pedro Gimenez | Increased concentrations of esters and fatty acids | Maturano et al. (2015) |
| *Debaryomyces vanrijiae* | Sequential | Pilot-scale | Muscat of Frontignan | Geraniol increase. High levels of β-glucosidase activity through fermentation. | García et al., 2002 |
| *Debaryomyces pseudopolymorphus* | Single/Co-inoculation | Lab-scale | Chardonnay | Mixed fermentations increase geraniol, nerol and citronellol content. | Cordero-Otero et al. (2003) |
| *Kazachstania aerobia* | Sequential | Medium-scale | Sauvignon Blanc | High ethyl acetate production. | Beckner Whitener et al. (2016) |
| *Kazachstania gamospora* | Sequential | Lab-scale/ Pilot-scale | Ribolla Gialla | Acetate and ethyl ester increase. | Dashko et al. (2015) |
| *Kazachstania gamospora* | Single | Lab-scale | Sauvignon blanc  and Syrah | Over 200 times more of phenethyl propionate was found in the musts fermented with *K. gamospora* compared to *S. cerevisiae* (control). | Beckner Whitener et al. (2015) |
| *Wickerhamomyces anomalus* | Sequential | Pilot-scale | Mazuela | Increased concentration of acetate- and ethyl- esters and panel preferences. | Izquierdo-Canas et al. (2014) |
| *Williopsis saturnus* | Co-inoculation | Lab-scale | Emir | The concentrations of acetic acid, ethyl acetate and isoamyl acetate were found higher in mixed culture experiments compared to control fermentation (*S. cerevisiae* alone). | Erten and Tanguler (2010) |
| *Zygotorulaspora florentina* | Co-inoculation | Lab-scale/Industry-scale | Sangiovese | An enhancement of polysaccharides and 2-phenylethanol content and a reduction of volatile acidity. High concentration of glycerol and esters. Higher ﬂoral notes. Low perception of astringency. | Lencioni et al. (2016) |
